# An Active Power Control Technique for Downlink Interference Management in a Two-Tier Macro–Femto Network

**DOI:** 10.3390/s19092015

**Published:** 2019-04-29

**Authors:** Tehseen Ul Hassan, Fei Gao

**Affiliations:** School of Information and Electronics, Beijing Institute of Technology, Beijing 100081, China; gaofei@bit.edu.cn

**Keywords:** active power control, Signal-to-Interference-plus-Noise-Ratio, inter-cell interference, femto user equipment, macro user equipment, path loss, green Impact and CO_2_ emission

## Abstract

The femtocell has evolved as a great solution for improving coverage and traffic offloading from the current LTE cellular networks, and it accomplishes the dreams of the high data rate for indoor mobile users. However, the exponentially expanding LTE femtocells cause interference in the network, as they share the same licensed spectrum with a macrocell. To tackle this issue, numerous interference mitigation techniques have been proposed in the literature. In this paper, we proposed an Active Power Control (APC) technique, which not only reduces Inter-Cell Interference (ICI) in a Macro User Equipment (MUE), generated from the downlink transmission power of an inadequately deployed femtocell, but also reduces unnecessary power consumption to achieve a green femtocell network. The simulation results show that the proposed APC technique effectively reduces ICI and optimizes the throughput performance of the MUE. Compared with the existing power control techniques, the APC technique provides a balanced trade-off in attaining necessary Quality-of-Service (QoS) of the Femto User Equipment (FUE) and reducing ICI to the victim MUE existing in the close proximity of the femtocell.

## 1. Introduction

The rapidly expanding demand for higher bandwidth, coverage and data rate in the mobile network has been a great stimulant to develop system capacity. The overlaid femtocell over macrocell in the heterogeneous network (HetNet) is a promising technology to accomplish the dreams of greater coverage, high efficiency and enhance the capacity of the cellular network. Femtocell is an energy efficient base station also called Femto Base Station (FBS) [1]. The femtocells are specifically designed for an indoor environment to enhance the capacity and quality having coverage of 10–20 m, named femto access point (FAP) [2]. The FAP shares the same licensed frequency spectrum with macrocell as it connected to the mobile operator’s network using public infrastructure such as broadband internet backhaul [3]. Traffic offloading from the LTE cellular network is currently a widely used method to manage exponentially increasing data traffic [4]. A femtocell is a promising candidate in this regard, as 45% of the mobile data traffic is offloaded through the femtocell because most of the data traffic originates from the indoor environment [5]. It is expected that, in the near future, indoor environments like schools, airports, homes, and offices will generate 60% and 90% of voice traffic and data traffic, respectively [6]. Although femtocells are deployed in the existing macrocell network to enhance network coverage in an indoor environment, the User Equipments (UEs) connected to them might be indoor or outdoor, depending on their location [7].

One of the attractive features of the femtocell is the access style. Three kinds of access control modes exist for the femtocell, i.e., closed, hybrid and open access modes. In the closed access mode, no other user can access femtocell services except for a Closed Subscriber Group (CSG) [8]. In the hybrid access mode, the CSG is prioritized over all other users, whereas in open access mode, all users i.e., OSG (Open Subscriber Group) can enjoy the service of the femtocell [9,10]. The access control mode plays an important role in terms of interference, i.e., the Inter-Cell Interference (ICI) is worse in the CSG mode [11].

The overlaid femtocell over macrocell in the two-tier heterogeneous network, where anmacrocell base station (also known eNB), works as a primary system and the Femto Base Station (also known as HeNB) as a secondary system. The two systems share the same frequency spectrum and work in a frequency reuse fashion [12]. The deployment of the femtocell has a massive role to manage interference in the two-tier network [13]. Inadequately deployed femtocell by users has lack of coordination with the macrocell. Normally the operator does not control such femtocells. The inter-cell interference becomes worse when the user deploys the femtocell in an ad-hoc style and moves it from one place to another. This can dramatically deteriorate the performance of the whole system by causing interference to cell edge Macro User Equipment (MUE) [14].

High transmission power of the femtocell enhances indoor coverage area and better signal strength; on the contrary, it causes a great amount of interference to the victim MUE existing in the coverage area of the femtocell. The interference is much worse when the MUE has the lower signal strength from the macrocell as compared to signal power from the femtocell [15]. This type of interference causes severe QoS degradation of the system, packet loss, transmission delay, and communication link failure. Besides the interference, high transmission power increases the atmospheric Carbon Dioxide (CO${}_{2}$) concentrations. The findings in the [16], show that the Information and Communication Technology (ICT) produces around 830 million tons of CO${}_{2}$ every year and is expected to double by 2020, which means more energy waste since carbon emission is a straightforward result of energy usage in the world, especially when we know that the main components of heterogeneous networks, which are the Macro Base Stations (MBSs) and FBSs, since the number of these stations in every country is growing exponentially. This means the rising demand of energy consumption increases carbon emission in the environment [17]. Thus, HeNB power should be carefully tuned for reducing the CO${}_{2}$ emissions but still maintaining QoS of the user. In this scenario, adaptive, active and cognitive resources management techniques can be the advanced solution rather than conventional resources management and customary cell planning [18,19].

In this paper, we proposed an Active Power Control (APC) technique, which not only reduces ICI to the victim MUE, generated by the downlink transmission power of the abysmal set up of femtocell, but also reduces unnecessary power losses by actively tuning its downlink transmission power. Although femtocells are low powered base stations, these power savings are crucial for the green deployment of femtocells, especially for dense deployment of femtocells that would result in increasing the total network consumption as millions of femtocells are expected to be deployed in the next few years. The simulation results show that the proposed APC technique effectively reduces the ICI and optimizes the throughput performance of MUE. Compared with the power control techniques based on the objective Signal-to-Interference-plus-Noise-Ratio (SINR) of Femto User Equipment (FUE), the MUE-assisted power control technique and range-based power control technique, the APC technique provides a balanced trade-off in attaining necessary Quality-of-Service (QoS) for FUE and mitigation of ICI to the macro user existing in the close proximity of the femtocell.

The rest of the paper is arranged in the following sections. Related work and the system model are discussed in Section 2 and Section 3, respectively. In Section 4, we discussed the existing power control techniques, whereas we presented the proposed Active power Control Technique (APC) in Section 5. We analyze the performance of the proposed and the existing approaches by conduction of simulation in Section 6. Finally, in Section 7, we draw a conclusion.

## 2. Related Work

Recently, numerous techniques have been presented in the literature to control downlink transmission power of the femtocell. In [20], a distributed joint resource allocation algorithm has been proposed to mitigate ICI in the two-tier femto-macro network. This algorithm consists of spectrum sensing, transmission mode selection and channel state information (CSI) estimation. The cognitive femtocell senses the unused slots and assigns a set of slots for each user. The slot allocation works is well in conjunction with power allocation centred on geometric programming to enhance the overall performance of the system. In [21], the proposed algorithm gradually reduces the downlink transmission power, whenever they informed about a cell edge macrocell users getting interference from its transmission power. This interference avoidance technique works when it receives information about MUE in the vicinity of the femtocell. Interference avoidance technique enables to deploy multiple femtocells in an indoor environment with higher capacity. The author presented cross-tier signal-to-leakage-plus-noise (SLNR) based Water Filling (CSWF) power allocation algorithm [22] for the mitigation of interference from the femtocell to the macrocell users. Cross-tier SLNR method reduces the major part of cross-tier downlink interference by using a modified Water Filling (WF) power allocation algorithm to determine the transmission power for each Resource Block (RB) of the femtocell. Cognitive power control technique in [23] used to address the interference problem from a femto base station to the nearby macrocell users. The FBS has the ability to sense its surrounding in terms of spectrum sensing and obtain the required information about downlink radio resources of a macrocell user. The FBS then tunes its transmission power efficiently and enhances the system capacity by mitigating interference. In [2], the Stochastic Approximation (SA) algorithm for downlink power control based on the information received through macrocell signalling. The femtocell then updates its downlink transmission power based on this information. The adjustment of Downlink (DL) power is based on the Channel Quality Indicator (CQI) and the ACK/NAK signal. A dynamic power tuning technique presented in [24] is used to tackle the downlink interference from HeNB to MUE. The HeNB tunes its downlink power based on information received from the interfered MUE. This interference mitigation technique helps to reduce the consumption of power and enhance the throughput of MUE. The MUE exists in the close proximity of HeNB, and receives interference from HeNB, to reduce such interference; a power control scheme proposed in [25] based on network listening. In [26], a novel scheme is proposed for interference mitigation in downlink cognitive femtocell networks, which is called joint channel allocation and power assignment. This scheme aims to reduce interference from the femtocell to MUEs and co-tier interference by collaboratively allocating power resources and channels between several femtocells; it depends on the Physical Cluster (PC) and the Virtual Cluster (VC). The writer also presented subcarrier power allocation and a VC-based power budget adjustment algorithm and Hungarian algorithm to better allocation of resources, minimize both co-tier and cross-tier interference in the femtocell. In [27], distributed coordination techniques in the DL of macrocells for controlling ICI caused by a femtocell in two-tier networks, where opportunistically reuse resources is an inherent requirement. The technique emphasis is on the autonomous operation of femtocell by self-organizing deployment. In [28], the downlink power control scheme presented to reduce the interference caused by a femtocell to its neighboring cell users. The femtocell will adjust the minimum transmit power on the basis of partial path-loss compensation which helps to reduce interference to adjacent cells and its users, that maintain the SINR level and assuages the required Quality-of-Service (QoS) of femtocell user. To minimize the interference caused by femtocells to MUEs in the downlink, a distributed Reinforcement Learning (RL) technique is used in [29], which is also called Distributed Power Control using Q-learning (DPC-Q). This technique identifies the sub-optimal pattern of power allocation in cognitive femtocell networks for increasing femtocell capacity. Independent Learning (IL) and Cooperative Learning (CL) approaches are applied to enhance performance.

## 3. System Model

The two-tier macro–femto heterogeneous network is where a macrocell and femtocell work as an essential primary system and secondary system, respectively, both systems share the same frequency (f = 2 GHz) spectrum [30]. $P_{M}$ represents the downlink transmission power of the eNB. Similarly, the HeNBs transmit with a fixed power $P_{F}$. In the LTE-Advanced (LTE-A) network, the system resources are divided along time slots and frequency sub-carriers. These resources are scheduled or distributed to users in units of Physical Resource Blocks (PRBs). We assume that the *K* PRBs are distributed uniformly among the MUEs in the macro-cell and the same resources are reused within each femtocell. In this paper, we consider an inadequately deployed HeNB in an indoor environment. The HeNB is linked to a macrocell through a broadband Internet backhaul link. The femtocell works in close access mode, where only CSG users can subscribe the services of the femtocell. The access styles of HeNB plays an important role in dealing with the interference. The interference is much sever in the closed access mode. Therefore, we consider the CSG mode to take the worst interference scenario into account.

**Figure 1:**
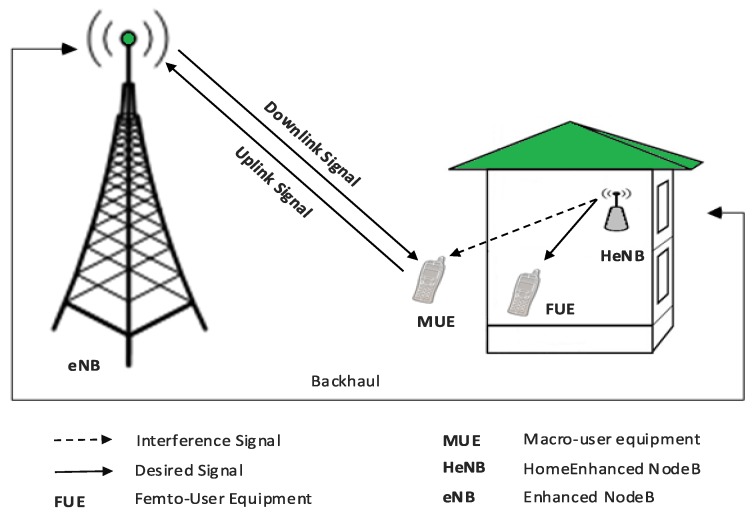
Interference scenario in HeNB and nearby macro and femto users.

Figure 1 shows the interference scenario of inter-cell interference (ICI) between the elements of the primary and secondary systems. In this case, the downlink link transmission of HeNB is the source of interference to MUE existing in its close proximity. We adopted the 3GPP LTE-A pathloss model for urban deployment of the femtocell [31].

### 3.1. Pathloss Model

The reduction of the power density of radio propagation from the Macro Base Station (MBS) to UE (indoor and outdoor) described in terms of pathloss calculated according to 3GPP LTE-Advanced pathloss model for urban deployments [32], which is given as.

(1) $$PL_{M}\left(dB\right)=\left\{\begin{array}{cc} 15.3+37.6log_{10}\left(R_{1}\right)+L_{p} & \left(Indoor\right)\\ 15.3+37.6log_{10}\left(R_{1}\right) & \left(Outdoor\right) \end{array}\right.$$

The $PL_{M}$ denoted as pathloss from the macrocell to the User equipment, $R_{1}$ is the range from the MBS to UE whereas the $L_{p}$ is the penetration loss through walls.

Similarly, the path loss from the femtocell to MUE and FUE existing in the close proximity (indoor and outdoor) can be calculated by using the path model as follows:

(2) $$PL_{F}\left(dB\right)=\left\{\begin{array}{cc} 38.46+20log_{10}\left(R_{2}\right) & \left(Indoor\right)\\ max\left(15.3+37.6log_{10}\left(R_{2}\right),38.46+20log_{10}\left(R_{2}\right)\right)+L_{p} & \left(Outdoor\right) \end{array}\right.$$

The $PL_{F}$ denoted as pathloss from the femtocell to the User Equipment. Similarly, the $R_{2}$ is the distance from HeNB to indoor and outdoor users.

### 3.2. Spectral Efficiency

In this paper, the spectral efficiency estimated by using Truncated Shannon Bound (TSB). The TBS has been used in 3GPP to optimally approximate the actual throughput by treating it as a function of SINR experienced by the user [32,33,34]. The spectral efficiency can be estimated as:

(3) $$Thr_{TBS}=\left\{\begin{array}{ccc} Thr=0 & For & SINR<SINR_{min}\\ Thr=B_{w}\alpha log_{2}\left(1+SINR\right) & For & SINR_{min}<SINR<SINR_{max}\\ Thr=Thr_{max} & For & SINR>SINR_{max} \end{array}\right.$$

In (3), bandwidth is denoted by $B_{w}$, the lower limit of SINR denoted by $SINR_{min}$ (throughput is zero below $SINR_{min}$) and upper limit denoted by $SINR_{max}$ (equal to the throughput of highest coding rate/modulation), whereas α is used to match the link level performance.

## 4. Power Control Techniques

### 4.1. Power Control Technique Assisted by FUE

In a real scenario, the HeNB instructs all the FUE to measure the received signal power from the neighboring interferer BS and send a feedback report [35]. The HeNB utilize the received information from the FUE and make necessary adjustments in downlink transmission power. The FUE-assisted power control technique (FUEAPCT) can be expressed as:

(4) $$P_{{FUE}_{Assisted}}=max\left(P_{min},min\left(P_{o},P_{max}\right)\right)$$

In (4), $P_{min}$ and $P_{max}$ is the minimum and maximum transmission power of the femtocell.

(5) $$P_{o}={P_{r}}_{FUE_{i}}+PL_{FBS\to FUE_{i}}\;+\;\varepsilon_{o}$$

Here, ${P_{r}}_{FUE_{i}}$ is the received power at $FUE_{i}$, $PL_{FBS\to FUE_{i}}$ is the pathloss from the FBS to the $FUE_{i}$. $\varepsilon_{o}$ is set to maintain the necessary QoS of femto users in (5).

### 4.2. Power Control Technique Assisted by MUE

The HeNB adjusts downlink transmission power based on the received information from the macro user through eNB backhaul, since there is no direct interface between MUE and the HeNB, the MUE sends measured information (interference message) to HeNB via the eNB backhaul. The HeNB utilizes the recived information from the MUE to tune its downlink transmission power. The MUE-assisted power control Technique (MUEAPCT) can be illustrated as:

(6) $$P_{{MUE}_{Assisted}}=max\left(min\left(\alpha P_{SINR}+\beta ,\;P_{max}\right)\;P_{min}\right)$$

In (6), $P_{SINR}$ define SINR between the macro user and the nearest femtocell, whereas α is a linear scalar that allows altering the slope of power control mapping curve, β is a parameter expressed in dB that can be used for altering the dynamic range of power control [35].

### 4.3. Range-Based Power Control Technique (RBPCT)

The Femtocell can adjust its downlink transmission power by considering the coverage area and the radius of both the macrocell and the femtocell; it also helps to maintain co tier-interference for inadequate deployment and dense femtocell networks [36]. The femtocell can dynamically tune its downlink transmission power by dividing the macrocell transmission power ($P_{M}$) over its coverage area ($X_{M}=log_{10}\left(R_{M}\right)$) and femtocell coverage area ($x_{F_{i}}=log_{10}\left(r_{F_{i}}\right)$), we obtained *Y* as follows:

(7) $$Y=\frac{P_{M}}{X_{M}}$$

In (7), the relationship between eNB downlink transmission power and its coverage area represented by *Y*. Consequently, it can be utilized to derive HeNB downlink power ($P_{i}$) as follows:

(8) $$P_{i}=Y\times x_{F_{i}}$$

Using Equation (8), Femtocell can adjust its downlink transmission power.

## 5. Proposed Active Power Control Technique

Figure 1 shows the ICI scenario between the elements of the primary and the secondary system. The downlink transmission power of femtocell causes interference to the MUE that existing in its coverage area. In such condition, MUE starts the handover or cell re-selection process. Since the femtocell is working in closed access mode, the macro user cannot subscribe to the services of the femtocell. In order to deal with such a scenario, the MUE measures the interference level from the HeNB and sends an interference message (IM) to the femtocell based on the Interference Indication Function (IDF) [37]. The IDF determines whether the interference experienced by the macro user is higher or lower than the threshold interference level. The IDF can be expressed as.

(9) $$I_{i}=P_{t}^{i}\psi_{i}{\left(R_{i}\right)}^{-\beta }$$

(10) $$x_{i}=\left\{\begin{array}{cc} 0, & I_{i}⩽I_{Threshold}\\ 1, & Otherwise \end{array}\right.$$

In (9), $P_{t}^{i}$ is the transmit power of the femtocell $F_{i}$. ψ represents log-normal shadowing, whereas the path loss is given as ${\left(R\right)}^{\beta }$. The range from the interfered FBS to UE is denoted as *R*, whereas the path loss component for the indoor transmission is β. In (10), $I_{Threshold}$ is the interference threshold set for the macro user. The IDF determines whether the interference experienced by the macro user is higher than the threshold interference to maintain the desired QoS of the MUE or the interference is lower than the threshold level. Based on the IDF, the macro user decides to send the interference message (IM) including HeNB information to eNB. When the femtocell receives the IM from the macro user via eNB backhaul, it understands that the non-CSG user is getting interference from its downlink transmission.

**Figure 2:**
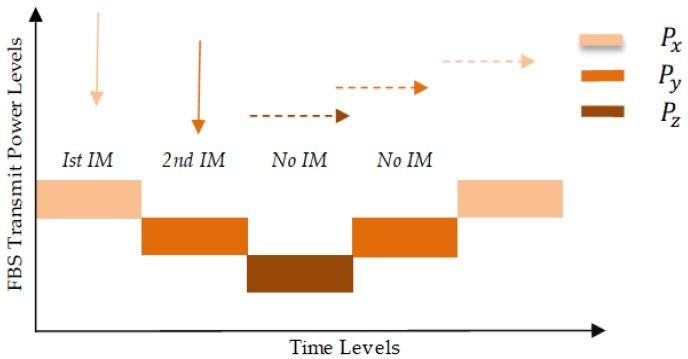
Femtocell transmit power tuning ($P_{x}$, $P_{y}$ and $P_{z}$).

In the first stage of the active power control technique, we introduced different power levels and time levels to tune the downlink power of the HeNB. The power levels ($P_{x}$, $P_{y}$ and $P_{z}$) and time levels ($TL_{1}$ and $TL_{2}$) were used to reduce and enhance transmission power based on received IM. The APC technique activates when the femtocell receives an IM from the MUE, The femtocell then takes necessary measures to tune its downlink transmission power from $P_{x}$ to $P_{y}$ (reduces $\Delta_{down}$). The time levels play an important role in the smooth tuning of HeNB transmission power. If the femtocell receives a new IM from the nearby MUE, it would not reduces its transmission power to $P_{z}$ level until the time level ($TL_{1}$) expires. Similarly, when HeNB has no IM and $TL_{1}$ expires, then the time level $TL_{2}$ starts and the transmission power level of the femtocell increases from $P_{z}$ to $P_{y}$ ($\Delta_{up}$). The same procedure of reducing and increasing transmission power will be carried out based the HeNB receiving a new interference message. Figure 2 show the femtocell adjusts its transmission power to respond to the IM from the MUE under the active power control technique.

The femtocell updates it downlink transmission power based on IM using following formulas

(11) $$\begin{array}{cc} P_{t}=P_{x} & No\;Interference\;Message \end{array}$$

(12) $$\begin{array}{cc} P_{t}=P_{y}=P_{x}-\Delta_{down} & Interference\;Message\;\&\;TL_{1}\;starts \end{array}$$

(13) $$\begin{array}{cc} P_{t}=P_{z}=P_{y}-\Delta_{down} & New\;Interference\;Message\;\&\;TL_{1}\;running \end{array}$$

(14) $$\begin{array}{cc} P_{t}=P_{y}=P_{z}+\Delta_{up} & No\;Interference\;Message\;\&\;TL_{2}\;starts \end{array}$$

(15) $$\begin{array}{cc} P_{t}=P_{x}=P_{y}+\Delta_{up} & No\;Interference\;Message\;\&\;TL_{2}\;running \end{array}$$

The IDF only focuses on the interference level of MUE. In order to maintain the necessary QoS of the FUE, we used the QoS Indication Function (ξ) in the second step, which indicates the minimum required QoS of FUE. The QoS Indication Function can be expressed as follows:

(16) $$\xi =\frac{P_{ref}\;\Gamma_{FUE}}{minRSRP_{j}}$$

In (16), $\Gamma_{FUE}$ is the minimum required SINR for FUE. $P_{ref}$ is the downlink reference signal transmit power of the HeNB. $minRSRP_{j}$ is the Reference signal received power measured by the $FUE_{j}$.

Using the above-described statement, the femtocell then effectively tunes its downlink power as:

(17) $$P_{APC}=max\left(P_{min},min\left(\xi \;P_{t},P_{max}\right)\right)$$

In (17), $P_{max}$ and $P_{min}$ are the maximum and minimum transmit power respectively.

Based on the aforementioned information, the received downlink SINR of the macro user equipment on PRB *k*(k=1,⋯,K) can be calculated as:

(18) $$SINR_{MUE}^{k}=\frac{P_{M}^{k}PL_{M}}{\sum_{i=1}^{F}x_{i}I_{i}+\sum_{F}P_{F_{APC}}^{k}PL_{F}+\sum_{M^{\prime }}P_{M^{\prime }}^{k}PL_{M^{\prime }}+P_{n}}$$

In (18), $P_{M}^{k}$ and $PL_{M}$ are the transmit power and pathloss of the serving eNB respectively. $P_{F_{APC}}^{k}$ is the transmit power of the interfering HeNB under the proposed power control scheme, whereas $PL_{F}$ is the pathloss from the interfering HeNB to UE. Similarly, $P_{M^{\prime }}^{k}$ and $PL_{M^{\prime }}$ are the transmits power and path loss of the interfering eNB to UE respectively. $P_{n}$ is the thermal noise density.

The received SINR of the Femto user equipment using the same analysis can be expressed as

(19) $$SINR_{FUE}^{k}=\frac{P_{F_{APC}}^{k}PL_{F}}{\sum_{i=1}^{F}x_{i}I_{i}+\sum_{M}P_{M}^{k}PL_{M}+\sum_{F^{\prime }}P_{F^{\prime }}^{k}PL_{F^{\prime }}+P_{n}}$$

In (19), $P_{F_{APC}}^{k}$ and $P_{F^{\prime }}^{k}$ are the transmission power of the serving and interfering femtocell respectively. $PL_{F^{\prime }}$ is the pathloss from the interferering HeNB to the UE.

Comparing with the existing techniques the proposed technique has the following advantages.

1. The Active Power Control Technique effectively reduces the inter-cell interference and optimize the throughput performance of the MUE.
2. The proposed technique not only reduces ICI to MUE but also maintains the QoS of FUE by considering the RSRP (Reference Signal Received Power) feedback from the Femto user to adjust its downlink transmission power.
3. The femtocell actively tunes its downlink power by using the power levels ($P_{x}$, $P_{y}$ and $P_{z}$) and time levels ($TL_{1}$ and $TL_{2}$), Hence, the proposed APC approach reduce the unnecessary power consumption to achieve green femtocell network.
4. Compared with existing power control approaches, the proposed approach offers significantly better performance in terms of downlink throughput CDF of the macro user and the femto user, the average throughput, FBS Power consumption and the green impact and CO${}_{2}$ emission.

The outlines of the proposed method are summarized in the Table 1.

## 6. Simulation Results and Discussion

We conducted extensive numerical experiments in MATLAB according to simulation assumptions and parameters used in 3GPP [38]. Table 2 shows a few summarized parameters. For simplicity, we have considered the widely used full buffer traffic model. It is characterized by the fact that the UE always has data to transmit or receive in the full buffer traffic model. The simulated interference scenario is shown in Figure 1. We assumed the dense urban deployed HeNB located in 25×25 house at the boundary of the centered eNB. The radius of eNB and HeNB is set to 500 m and 25 m, respectively. The eNB has a maximum transmitting power of 43 dBm. Similarly, the HeNB has 21 dBm and 0 dBm maximum and minimum transmitting power respectively. In this simulation, the close access mode is considered, which is the most favorite mode of indoor users, in which the CSG users enjoy higher data rate and capacity and non-CSG users are not allowed to use the femtocell services. The interference is severe in closed access mode as compared to other access modes.

In this paper, we conducted the simulation with “No Power Control or Fixed Power Control Technique (FPCT)” as baseline, where there is no power control technique activated in the femtocell, therefore, the femtocell transmits maximal power. It is a fact that high transmission power of HeNB provides better signal strength, good coverage to the femto users; conversely, the cell edge macro users receive interference from it. Therefore, the interference technique operating in the femtocell should provide a balanced trade-off between the throughput of macrocell and femtocell users.

In the following subsections, we evaluated the performance of proposed and existing power control techniques in-terms of downlink throughput distribution, average throughput distribution, FBS power consumption and green impact and CO${}_{2}$ emission by conducting numerical experiments.

### 6.1. Analyzing Downlink Throughput Distribution

**Figure 3:**
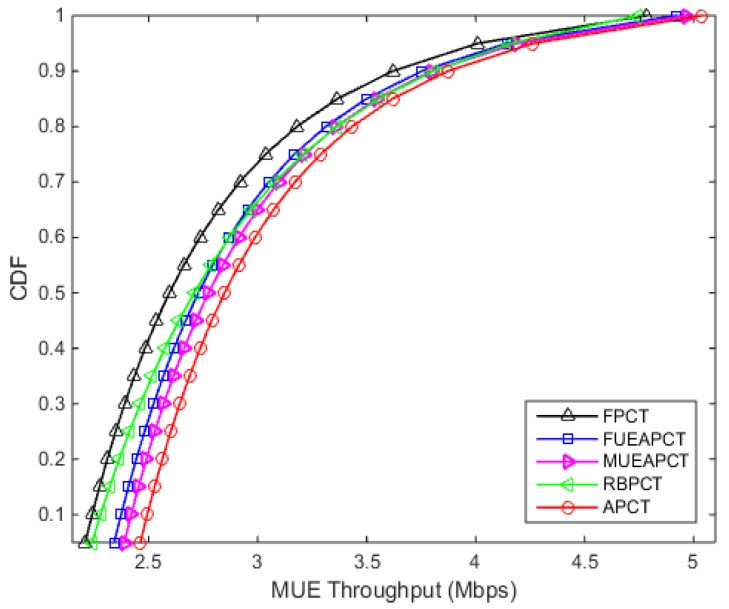
Downlink Throughput CDF of the Macrocell User.

The simulation results in Figure 3 show that the downlink throughput distribution of macro users, which is also called non-CSG users in this case. The downlink throughput of macro user drops severely when there is no power control technique activated in the femtocell. However, the proposed active power control technique outperforms its counterparts. It can be seen from the Figure 3, the macro user achieve value of CDF of throughput 2.90 Mbps at 0.5 or 50% under the proposed APC technique, at the same point the FPCT, FUEAPCT, MUEAPCT and RBPCT achieve 2.60 Mbps, 2.73 Mbps, 2.77 Mbps and 2.71 Mbps receptively.

**Figure 4:**
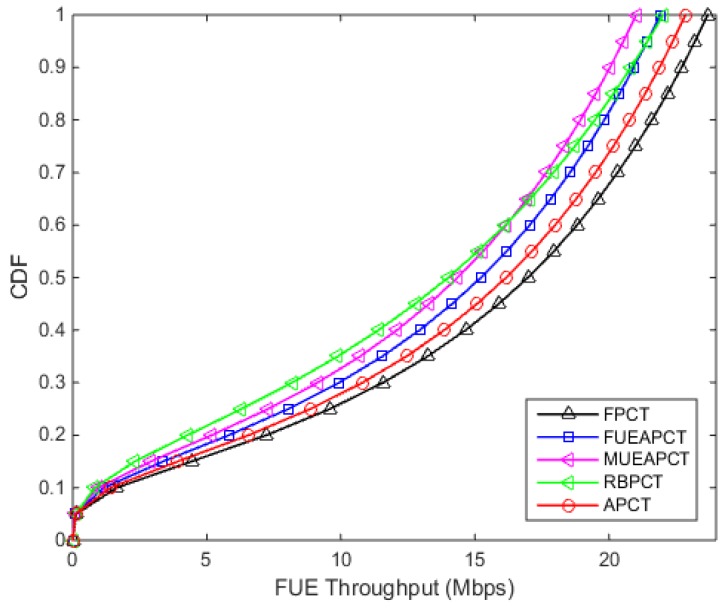
Downlink Throughput CDF of the Femtocell User.

Figure 4 shows the downlink CDF of the femto users. From the results, it can be seen that the proposed APC technique offers better performance except for ”No Power control approach”. With no power control, the HeNB transmits maximal power. Therefore, the femto users enjoy higher throughput at the cost of MUE performance. It can be seen from Figure 4 that the femto user achieves value of CDF of throughput 16.17 Mbps at 0.5 or 50% under the proposed APC technique, at the same point the FPCT, FUEAPCT, MUEAPCT and RBPCT achieve 16.98 Mbps, 15.23 Mbps, 14.36 Mbps and 14.01 Mbps receptively.

From the Figure 3 and Figure 4, we can easily see that the APC technique provide a balanced trade-off between the downlink throughput of macro users and femtocell users. In other words, this power control technique reduces the interference level to macro users while meeting the required QoS standard of the femto users.

### 6.2. Average Throughput

**Figure 5:**
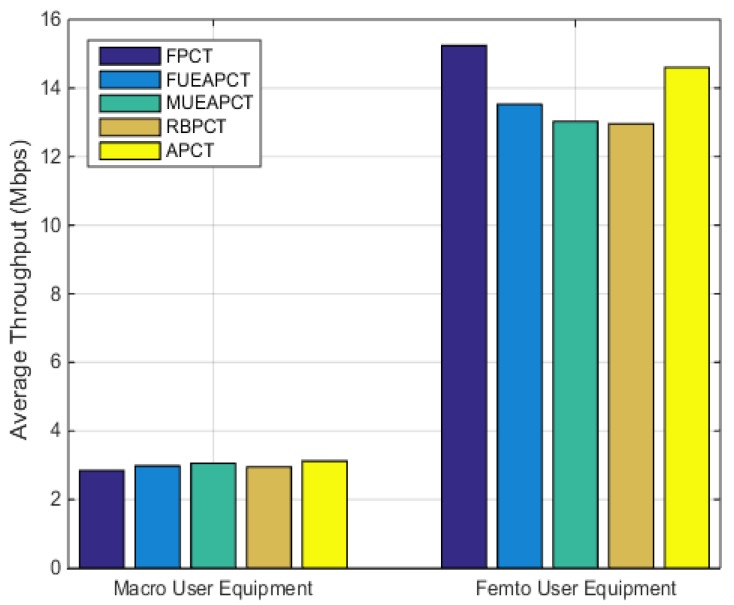
Average throughput under different power control approaches.

In this subsection, we presented a comparison graph of the average throughput of the MUE and FUE under FUE-assisted, MUE-assisted, range based power control technique and the proposed active power control technique in Figure 5. The graph indicates that the proposed power control provides a balanced trade-off between the performance of the femto user and the macro user.

### 6.3. FBS Power Consumption

**Figure 6:**
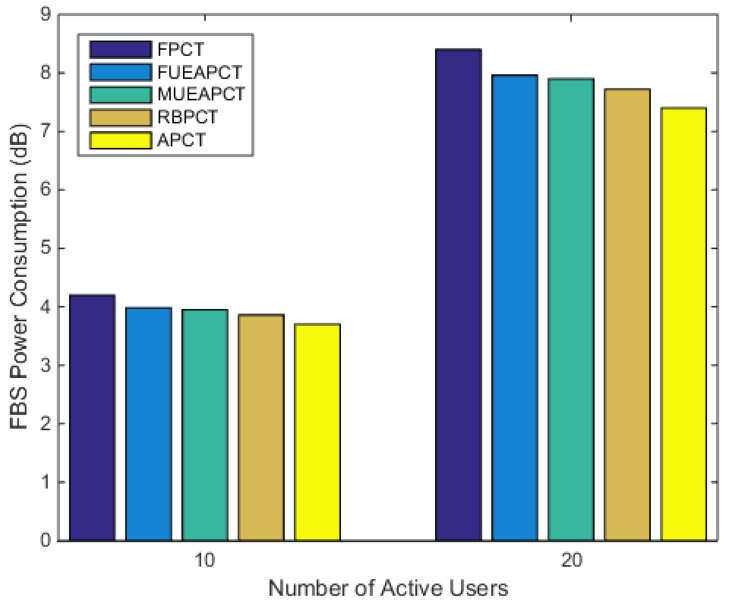
FBS Power Consumption Vs number of active subscribers.

In Figure 6, we plot the FBS power consumption against the number of the active subscribers. In this scenario, the femtocell transmits under the aforementioned power control techniques in a radio-hostile environment. In order to carry out a candid comparison, the number of assigned resource blocks for a user were kept constant. We can see from Figure 6 that the proposed active power control technique outperforms its counterpart in reducing unnecessary power losses. Under the same scenario, the APC approach consumed 13.51% less power than the baseline. Whereas, the FUEAPCT, MUEAPCT and RBPCT, respectively, consumed 7.5%, 6.39% and 4.9% less power as compared to the baseline.

### 6.4. Green Impact and CO${}_{2}$ Emission

The CO${}_{2}$ emission depends on the power supply in the base station. The important factor in the green impact and CO${}_{2}$ emission is the conversion factor ($c_{f}$). The value of CO${}_{2}$ emission conversion factor is different for the different energy sources [39]. The total CO${}_{2}$ emission can be calculated as follows:

(20) $${\mathrm{CO}}_{2}\;\mathrm{Emission}\;\left(\mathrm{kg}\right)=\mathrm{Power}\;\left(\mathrm{kWh}\right)\times c_{f}$$

Based on the statistics in [40], the power consumption of a full load HeNB is assumed to be 165.42 kWh/year. The author in [41], assumed a constant CO${}_{2}$ emission conversion factor of 0.5 kg CO${}_{2}$-e/kWh. Using the $c_{f}$, we can estimate that a full load HeNB under No Power control approach emits 82.71 kg of CO${}_{2}$ per year. Table 3, shows the estimated CO${}_{2}$ saving per year for one femtocell deployed in the dense urban area. From the Table 3, we can see that the proposed active power control technique offer better performance compared to its counterpart by saving 11.17 kg of CO${}_{2}$ emission per year.

The aim of the proposed APC technique is not only reducing ICI to MUE in close proximity of the femtocell and maintaining necessary QoS of FUE, but also reduces the unnecessary power losses. Moreover, the APC techniques activate only when the femtocells receive an IM from the victim MUE. The IM originates on the basis of IDF when the interference from the femtocell to victim MUE is higher than that of threshold interference. Before tuning its downlink transmission, HeNB seeks for the RSRP feedback report from the femto user. On the basis of IM and feedback report, HeNB actively tunes it downlink transmission power, whereas in proactive technique, the HeNB does not take care of the existence of nearby victim MUE and it tunes its downlink transmission power based on RSRP (Reference Signal Received Power), therefore, the proposed APC reduces unnecessary power consumption. Although femtocells are low-powered base stations, these power savings are crucial for the green deployment of femtocells, especially for dense deployment of femtocells that would result in increasing the total network consumption as millions of femtocells are expected to be deployed in the next few years.

## 7. Conclusions

In this paper, we proposed an APC technique to scale down the ICI level in the macro user while maintaining the necessary QoS of the femto user. This type of interference is caused by the downlink transmission of inadequately deployed femtocell. The proposed APC technique activates when the macro user sends an IM based on IDF to HeNB through the backhaul link. The HeNB then seeks for the RSRP feedback report from the FUE. Based on the IM and the feedback report, HeNB actively tunes its downlink power using different power levels and time levels, therefore, it reduces unnecessary consumption of power by saving 13.51% with respect to the baseline. The analysis of the green impact and CO${}_{2}$ emission show that the proposed APC technique saved 11.17 kg/year of CO${}_{2}$ emission for one full load HeNB, which is higher than the existing power control techniques. The simulation results have demonstrated that the proposed APC technique effectively reduces the inter-cell interference (ICI) and optimizes the throughput performance of MUE. Compared with the power control techniques based on the objective Signal-to-Interference-plus-Noise-Ratio (SINR) of FUE, MUE-assisted power control technique and range based approach, the APC technique provides a balanced trade-off in attaining necessary Quality-of-Service (QoS) for FUE and mitigation of ICI to the nearby macro user.

## Figures and Tables

**Table 1:** Active Power Control Approach.

Input	Interference Messages and FUE Feedback Report.
Output	Downlink Transmission Power Tuning.
step 1	Using (10), the macro user decides to send IM including HeNB information to eNB.
step 2	HeNB instruct its users to send a feedback report. Based on the (16), which indicates the minimum required QoS, the FUE send the information to HeNB.
step 3	Based on the IM from the MUE and QoS indication report from the FUE, HeNB actively tune its downlink transmission power using (17)

**Table 2:** Simulation parameters.

Parameters	Assumptions
Carrier frequency (f)	2 GHz
Transmit power of macrocell $\left(P_{M}\right)$	43 dBm
HeNB Noise Figure	8 dB
Femtocell’s Transmit Power $\left(P_{F}\right)$	$P_{max}=21$ Bm and $P_{min}=0$ dBm
Lognormal shadowing standard deviation for Femtocell	4 dB
Macrocell coverage Area $\left(R_{1}\right)$	500 m
Shadowing standard deviation for Macrocell	8 dB
Femtocell Coverage Area $\left(R_{2}\right)$	25 m
Exterior wall penetration loss	5 dB
Interior penetration loss ($L_{p}$)	15 dB
Thermal Noise Density (η)	−174 dBm/Hz
Bandwidth $\left(B_{w}\right)$	10 MHz
Macrocell antenna Gain	14 dBi
Access Mode	CSG
Interference threshold, $I_{Threshold}$	−72 dBm
Minimum separation UE to HeNB	0.2 m
Minimum separation UE to eNB	35 m
$TL_{1}$ and $TL_{2}$	200 ms
$\Delta_{up}\;and\;\Delta_{down}$	2 dB
Traffic Model	Full Buffer
Minimum required SINR for FUE ($\Gamma_{FUE}$)	10 dB

**Table 3:** Green Impact Calculation and CO${}_{2}$ Emission.

Power Control Techniques	Power Saving (%)	CO${}_{2}$ Saving (kg/year)
FUE Assisted Power Control Technique	7.5	6.20
MUE Assisted Power Control Technique	6.39	5.29
Range Based Power Control Technique	4.9	4.05
Proposed Active Power Control Technique	13.51	11.17
